# Cord blood group 2 innate lymphoid cells are associated with lung function at 6 weeks of age

**DOI:** 10.1002/cti2.1296

**Published:** 2021-07-21

**Authors:** Gabriela Martins Costa Gomes, Patricia de Gouveia Belinelo, Malcolm R Starkey, Vanessa E Murphy, Philip M Hansbro, Peter D Sly, Paul D Robinson, Wilfried Karmaus, Peter G Gibson, Joerg Mattes, Adam M Collison

**Affiliations:** ^1^ Priority Research Centre GrowUpWell® – Hunter Medical Research Institute The University of Newcastle Newcastle NSW Australia; ^2^ Priority Research Centre for Healthy Lungs ‐ Hunter Medical Research Institute University of Newcastle Newcastle NSW Australia; ^3^ Department of Immunology and Pathology Central Clinical School Monash University Melbourne VIC Australia; ^4^ Centenary UTS Centre for Inflammation Centenary Institute Sydney NSW Australia; ^5^ Child Health Research Centre University of Queensland Brisbane QLD Australia; ^6^ Department of Respiratory Medicine The Children's Hospital at Westmead Sydney NSW Australia; ^7^ School of Public Health University of Memphis Memphis TN USA; ^8^ Sleep Medicine Department John Hunter Hospital Newcastle NSW Australia; ^9^ Paediatric Respiratory & Sleep Medicine Department John Hunter Children's Hospital Newcastle NSW Australia

**Keywords:** asthma, cord blood, CRTh2 ILC2, multiple breath washout, tidal breathing

## Abstract

**Objective:**

Offspring born to mothers with asthma in pregnancy are known to have lower lung function which tracks with age. Human group 2 innate lymphoid cells (ILC2) accumulate in foetal lungs, at 10‐fold higher levels compared to adult lungs. However, there are no data on foetal ILC2 numbers and the association with respiratory health outcomes such as lung function in early life. We aimed to investigate cord blood immune cell populations from babies born to mothers with asthma in pregnancy.

**Methods:**

Cord blood from babies born to asthmatic mothers was collected, and cells were stained in whole cord blood. Analyses were done using traditional gating approaches and computational methodologies (t‐distributed stochastic neighbour embedding and PhenoGraph algorithms). At 6 weeks of age, the time to peak tidal expiratory flow as a percentage of total expiratory flow time (tPTEF/tE%) was determined as well as Lung Clearance Index (LCI), during quiet natural sleep.

**Results:**

Of 110 eligible infants (March 2017 to November 2019), 91 were successfully immunophenotyped (82.7%). Lung function was attempted in 61 infants (67.0%), and 43 of those infants (70.5% of attempted) had technically acceptable tPTEF/tE% measurements. Thirty‐four infants (55.7% of attempted) had acceptable LCI measurements. Foetal ILC2 numbers with increased expression of chemoattractant receptor‐homologous molecule (CRTh2), characterised by two distinct analysis methodologies, were associated with poorer infant lung function at 6 weeks of age.”

**Conclusion:**

Foetal immune responses may be a surrogate variable for or directly influence lung function outcomes in early life.

## Graphical abstract

Cord blood CRTh2^high^ ILC2 populations from infants born to asthmatic mothers may influence lung function later in life. The association between cord blood CRTh2^high^ ILC2 and foetal eosinophils numbers may indicate a role of foetal ILC2 in promoting eosinophilia, which warrants further investigation.

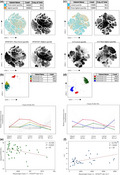

## Introduction

During pregnancy, the maternal immune response is shifted towards a type 2 (T2) dominant response that promotes immunological tolerance towards the foetus. In pregnant women with asthma, the T2 response is further exaggerated, and interferon (IFN) production is lower, resulting in increased inflammatory responses and reduced antiviral immunity.^1^, ^2^ The foetal immune system is thought to be under the direct influence of the maternal T2 immune response mounted at the foetus–maternal interface. Infants who fail to develop a mature immune response in the first 6 months of life with a shift from T2 dominance towards immunological tolerance have the highest risk to develop allergic diseases in later life.^3^, ^4^

Innate lymphoid cells (ILCs) are a subset of immune cells with lymphoid morphology that lack antigen receptors and typical lineage markers.^5^ Group 2 ILCs (ILC2) denote the population of ILCs that produce T2 cell‐associated cytokines^6^ and are elevated in asthmatic patients in the lung, sputum and blood.^7^, ^8^, ^9^, ^10^ It is well established that ILC2 is a major cellular source of interleukin (IL)‐5 and IL‐13, during the initiation and maintenance of allergic lung disease,^11^ and is the primary source of IL‐5 within the lung.^12^ Employing IL‐5 transgenic mice, Lebold *et al*.^13^ showed that maternal IL‐5 crosses the placenta and causes eosinophilia in the foetus and stimulates foetal IL‐5 production that promoted vagally mediated airway constriction. It is plausible that the *in utero* promotion of lung eosinophilia induced by IL‐5‐producing ILC2 may increase sensory innervation of the airways, wiring them for the development of subsequent airway hyperreactivity.^14^

Lung function is an objective parameter to assess respiratory outcomes very early in life, at a time when respiratory symptoms are yet to develop. Lung function parameters are sensitive to conditions and exposures adversely affecting the foetus, such as premature birth, *in utero* tobacco smoke exposure and maternal asthma in pregnancy.^15^, ^16^, ^17^ Environmental exposure to air pollution in childhood can increase the risk of developing asthma even in individuals with high lung function in infancy.^18^ While several studies have shown that infants with poor lung function at birth are at increased risk of the subsequent development of asthma, this has not been universally reported^19^, ^20^, ^21^, ^22^, ^23^ and further work is required to understand the nuanced relationship between lung function and asthma through childhood.^24^

The measurement of flow‐volume parameters during naturally occurring sleep in young infants' tidal breathing has been extensively used and validated.^25^, ^26^, ^27^ Time to peak tidal expiratory flow divided by the total time of tidal expiratory flow (tPTEF/tE%) is recognised as an integrated output of the entire respiratory system, including airflow limitation and control of breathing in young infants, and these values are reduced in the presence of airway obstruction.^26^ The multiple breath washout (MBW) technique is used as a sensitive marker to measure the efficiency of ventilation distribution in the lungs quantified as Lung Clearance Index (LCI).^28^ LCI represents the number of the functional residual capacity (FRC) volume turnovers required to wash out the tracer gas during testing with higher values indicative of lung ventilation inhomogeneities because of small airway constriction. Both tPTEF/tE% and LCI have been reported as being altered in infants and children with a range of respiratory conditions^29^, ^30^, ^31^ and are predictive of later respiratory health.^32^, ^33^

Considering the direct influence of maternal T2 immune response at the foetus–maternal interface that ILC2 is the primary innate source of T2 cell‐associated cytokines,^6^ which are elevated in asthmatic patients' blood,^7^ the relationship between ILC2 and lung function in early life was here explored. Cord blood was immunophenotyped as a representative snapshot of the *in utero* immune environment^34^ in conjunction with quantitative measurement of infant lung function. Since ILC2 has been reported to cluster in the vicinity of the airway epithelium and alveolar space,^35^, ^36^ tPTEF/tE% as well as LCI were measured to investigate multiple aspects of lung function in 6‐week‐old babies born to asthmatic mothers.

## Results

### Study population

**Figure 1:**
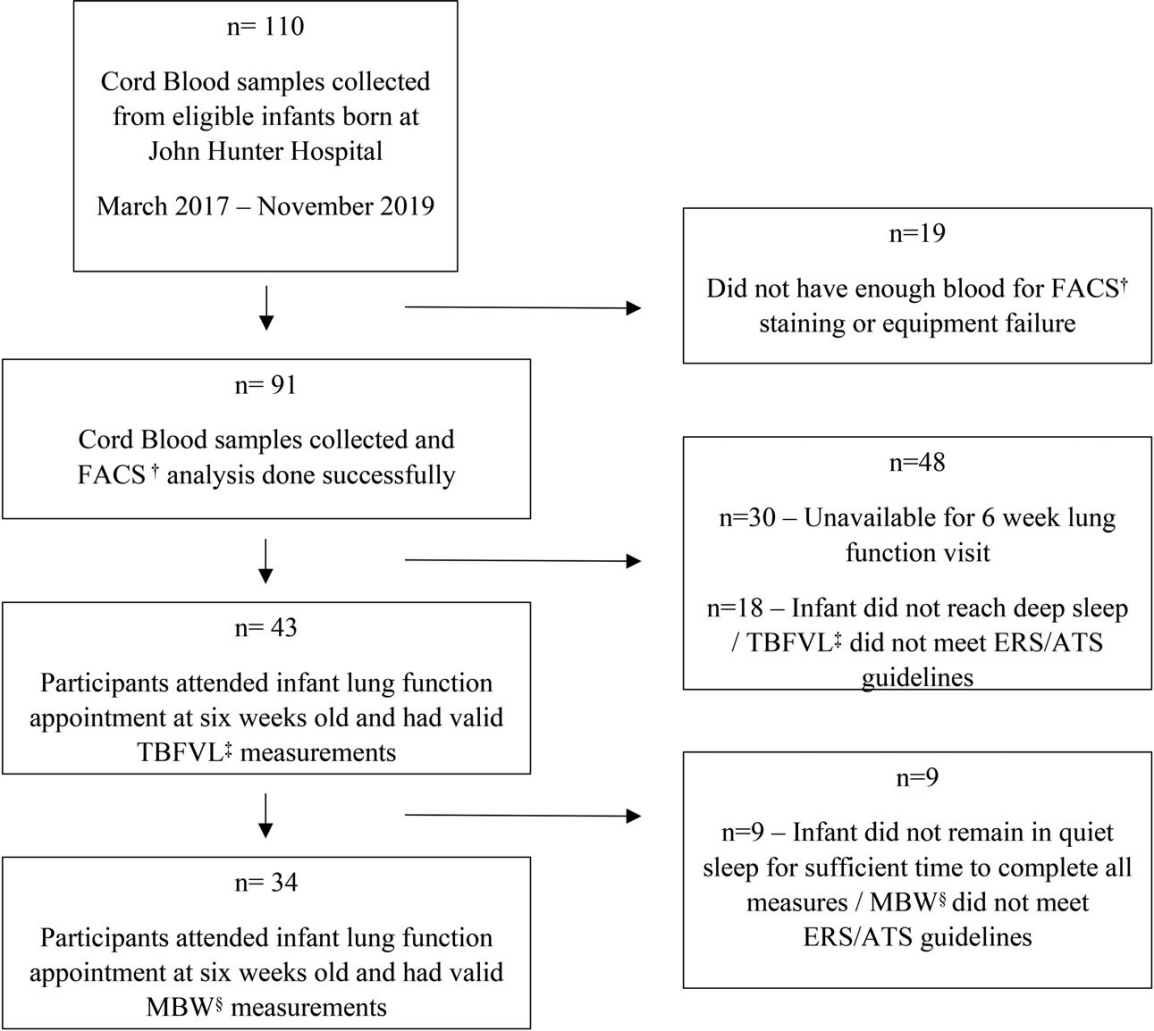
Flow chart. Recruitment, collection of cord blood samples and the success rate of lung function tests. FACS, fluorescence‐activated cell sorting; MBW, multiple breath washout; TBFVL, tidal breathing flow volume loop.

To explore associations between profiles of subsets of immune cells in the cord blood and lung function in early infancy, we enrolled babies born to asthmatic women, who had asthma in pregnancy and participated in the Breathing for Life Trial (BLT).^37^ A characteristic table of the subjects enrolled is presented in Table 1. All participants gave written informed consent (ref no. 12/10/17/3.04). Between March 2017 and November 2019, 110 eligible infants were born to mothers participating in BLT at John Hunter Hospital, Newcastle site. From those infants, 91 (82.7%) had cord blood collected immediately after birth and had cells stained in whole blood and subsets predefined based on specific surface markers (Supplementary table 1) within 6 h. Lung function was attempted in 61 infants (67.0%), and 43 of those infants (70.5% of attempted) had acceptable and successful tidal breathing flow volume loop (TBFVL) measurements while 34 infants (55.7% of attempted) had acceptable and successful sulphur‐hexafluoride (SF6) MBW tests at the same age (Figure 1). The percentage of cord blood cells stained were maintained between all groups analysed (Supplementary table 2).

**Table 1:** Characteristic table of subjects with cord blood collected and valid tPTEF/tE% and LCI tests at 6 weeks of age (results are shown as average)

	CB samples – FACS analysis *n* = 91	CB samples – FACS and tPTEF/tE% *n* = 43	CB samples – FACS and LCI *n* = 34
Gravidity (min–max)	2.6 (1–9)	2.6 (1–9)	2.6 (1–9)
Caesarean section (%)	33 (36.3)	13 (30.2)	8 (23.5)
Maternal age (min–max)	30.0 (19.0–41.5)	31.0 (20.0–40.8)	31.4 (20.2–40.8)
Male (%)	49 (53.8)	25 (58.1)	19 (55.9)
Maternal smoking (%)	11 (12.1)	3 (7.0)	2 (5.9)
Gestational age at delivery (min–max)	39.0 (34.0–41.0)	39.0 (37.0–41.0)	39.0 (37.0–41.0)
Birthweight (kg) (min–max)	3.5 (2.1–4.9)	3.6 (2.2–4.9)	3.6 (2.2–4.9)
Birth length (cm) (min–max)	51.6 (30.7–58.0)	52.4 (47.0–57.2)	52.2 (47.0–57.2)
Age at test (days) (min–max)		48.1 (33.0–73.0)	48.5 (33.0–73.0)
Length at test (cm) (min–max)		56.0 (50.0–62.0)	56.0 (50.0–62.0)
Weight at test (kg) (min–max)		5.0 (3.6–6.7)	4.9 (3.6–6.7)
Weight gain until test day (kg) (min–max)		1.35 (0.03–2.60)	1.33 (0.03–2.60)
Weight gain by day until test day (kg) (min–max)		0.03 (0.00–0.05)	0.03 (0.00–0.05)
Exclusive breastfeeding at test (%)		22 (51.2)	18 (51.4)
Mixed breastmilk and formula since birth (%)		15 (34.8)	12 (34.3)
Never breastfed (%)		6 (14.0)	4 (11.4)

CB, cord blood; FACS, fluorescence‐activated cell sorting; LCI, Lung Clearance Index; tPTEF/tE, ratio of time to reach peak tidal expiratory flow to total expiratory time.

### ILC2 and CRTh2^high^ ILC2 correlate with tPTEF/tE% and LCI

**Figure 2:**
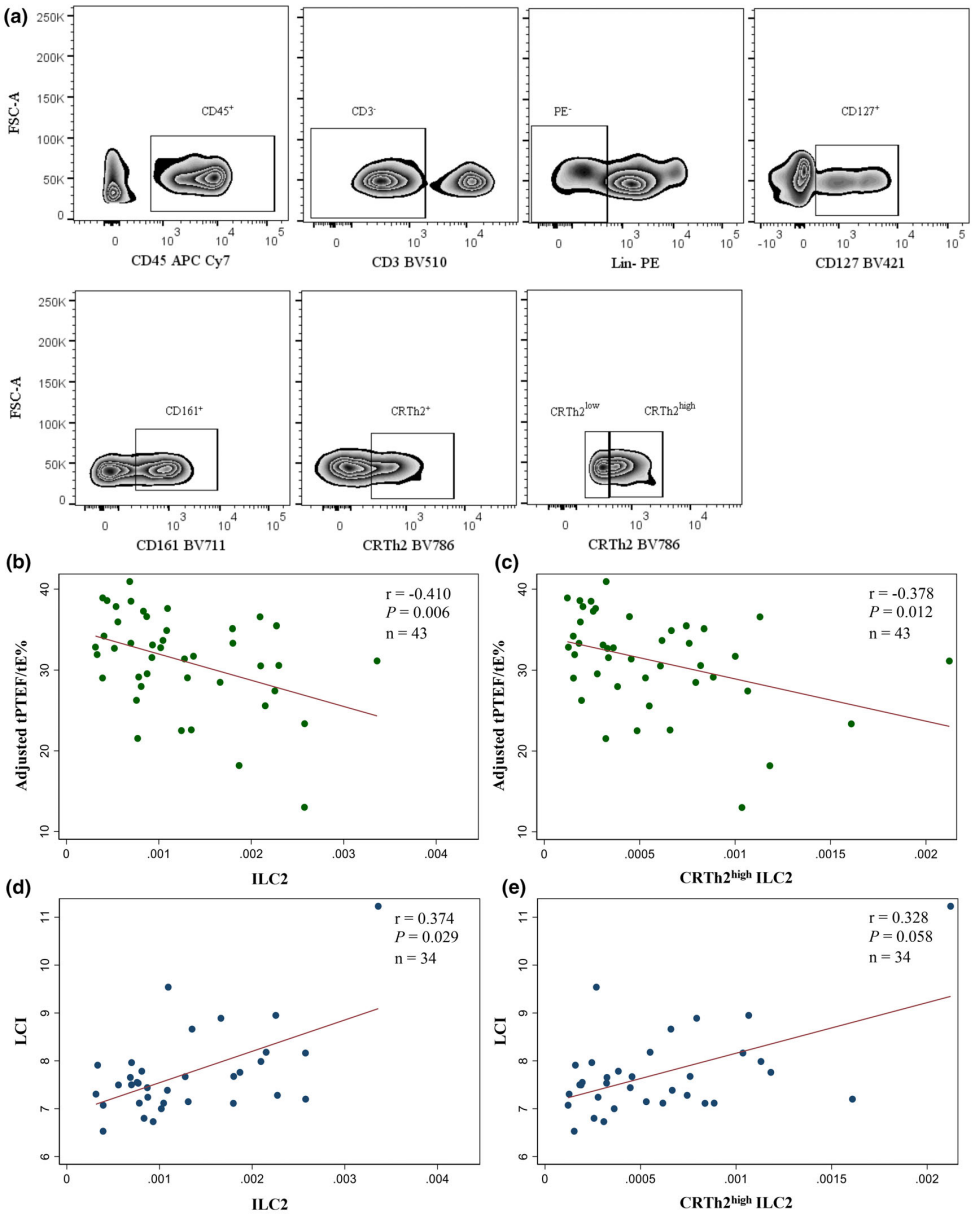
Cord blood ILC2 and its relationship with infant lung function. Cord blood samples stained and acquired with a LSRFortessa X‐20 flow cytometer and analysed using FlowJo software. Representation of flow cytometry gating strategy **(a)**. Pearson correlation analysis between adjusted tPTEF/tE% at 6 weeks of age and total ILC2 [CD45^+^ lineage^−^ (CD3, TCR‐αβ, TCR‐γδ, CD19, CD11c, CD94, CD14, CD1a, CD34, CD123, CD303, FceRIa) CD127^+^, CD161^+^, CRTh2^+^] **(b)** and CRTh2^high^ ILC2 (CD45^+^ lineage^−^ CD127^+^, CD161^+^, CRTh2^high^) **(c)** in cord blood (*n* = 43). Spearman correlation analysis between LCI at 6 weeks of age and total ILC2 **(d)** and CRTh2^high^ ILC2 **(e)** in cord blood, analysed by standard biaxial gating (*n* = 34).

Considering ILC2 analysed by the standard biaxial gating strategy (Figure 2a), correlation analyses were applied. tPTEF/tE% negatively correlated with total ILC2 (Figure 2b) as well as with CRTh2^high^ ILC2 (Figure 2c). Spearman analysis showed a positive correlation between LCI and total ILC2 (Figure 2d) and also between LCI and CRTh2^high^ ILC2 (Figure 2e). However, there was no correlation between normalised tPTEF/tE% and LCI (*r* = −0.237, *P* = 0.178). Thus, total ILC2 and CRTh2^high^ ILC2 numbers in cord blood were associated with impaired lung function and increased lung ventilation inhomogeneities.

### CRTh2^high^ ILC2 are predictors of tPTEF/tE% and LCI in a multivariable and univariable regression

Linear regression analyses were performed to determine the association between ILC2 numbers in cord blood and infant lung function. tPTEF/tE% was significantly associated with CRTh2^high^ ILC2 numbers [Table 2, beta‐parameter estimate −11.790, 95% confidence interval (CI) −20.695 to −2.885 and *P* = 0.011] in the multivariable regression analysis. Eosinophils (beta‐parameter estimate −0.001, CI −0.087 to 0.084 and *P* = 0.972), neutrophils (beta‐parameter estimate 0.020, CI −0.024 to 0.064 and *P* = 0.368), active CD4 T cells (beta‐parameter estimate 0.002, CI −0.028 to 0.031 and *P* = 0.897), active CD8 T cells (beta‐parameter estimate −0.007, CI −0.077 to 0.062 and *P* = 0.831), Treg (beta‐parameter estimate −0.167, CI −0.439 to 0.105 and *P* = 0.220), B cells (beta‐parameter estimate 0.002, CI −0.005 to 0.008 and *P = *0.581), NK cells (beta‐parameter estimate 0.002, CI −0.006 to 0.009 and *P* = 0.664), ILC1 (beta‐parameter estimate 15.504, CI −1.560 to 32.568 and *P* = 0.074), ILC2 total (beta‐parameter estimate −4.734, CI −10.133 to 0.664 and *P* = 0.084), CRTh2^low^ ILC2 (beta‐parameter estimate −3.410, CI −13.018 to 6.199 and *P* = 0.476) and ILC3 (beta‐parameter estimate −2.748, CI −12.945 to 7.450 and *P* = 0.588) were not significantly linked with normalised tPTEF/tE% (Table 2).

**Table 2:** Linear regression analysis used to identify variables associated with tPTEF/tE% and with LCI

tPTEF/tE%	Multivariable analysis^a^				LCI	Univariable analysis			
	Coefficient	SE	*P*‐value	95% CI		Coefficient	SE	*P*‐value	95% CI
Eosinophils	−0.001	0.042	0.972	−0.087; 0.084	Eosinophils	0.004	0.003	0.204	−0.002; 0.011
Neutrophils	0.020	0.022	0.368	−0.024; 0.064	Neutrophils	−0.002	0.002	0.197	−0.005; 0.001
Active CD4 T cells	0.002	0.015	0.897	−0.028; 0.031	Active CD4 T cells	−0.002	0.002	0.375	−0.005; 0.002
Active CD8 T cells	−0.007	0.034	0.831	−0.077; 0.062	Active CD8 T cells	−0.003	0.003	0.367	−0.010; 0.004
Treg	−0.167	0.134	0.220	−0.439; 0.105	Treg	−0.007	0.012	0.577	−0.032; 0.018
B cells	0.002	0.003	0.581	−0.005; 0.008	B cells	0.0001	0.0003	0.784	−0.001; 0.001
NK cells	0.002	0.004	0.664	−0.006; 0.009	NK cells	0.0003	0.0003	0.420	−0.0004; 0.001
ILC1	15.504	8.406	0.074	−1.560; 32.568	ILC1	0.312	0.680	0.649	−1.074; 1.698
ILC2	−4.734	2.659	0.084	−10.133; 0.664	ILC2	0.655	0.176	0.001	0.297; 1.013
CRTh2^high^ ILC2	−11.790	4.386	0.011	−20.695; −2.885	CRTh2^high^ ILC2	1.058	0.297	0.001	0.452; 1.663
CRTh2^low^ ILC2	−3.410	4.733	0.476	−13.018; 6.199	CRTh2^low^ ILC2	0.890	0.388	0.029	0.098; 1.681
ILC3	−2.748	5.023	0.588	−12.945; 7.450	ILC3	0.482	0.447	0.289	−0.429; 1.393

Coefficients relative to cells are expressed as the change in the lung function measure per 10^3^ of the identified cell type.

^a^ Multivariable analysis for each cell type adjusted for male sex, birth order, exclusive breastfeeding until test day, weight gain until test day and age at test (days).

In univariable regression, LCI was significantly associated with total ILC2 (beta‐parameter estimate 0.655, CI 0.297 to 1.013 and *P* = 0.001), CRTh2^low^ ILC2 (beta‐parameter estimate 0.890, CI 0.098 to 1.681 and *P* = 0.029) and also CRTh2^high^ ILC2 numbers (beta‐parameter estimate 1.058, CI 0.452 to 1.663 and *P* = 0.001; Table 2). Confirming that CRTh2^high^ ILC2 numbers in cord blood are associated with lung function at 6 weeks. Other cell populations – eosinophils (beta‐parameter estimate 0.004, CI −0.002 to 0.011 and *P* = 0.204), neutrophils (beta‐parameter estimate −0.002, CI −0.005 to 0.001 and *P* = 0.197), active CD4 T cells (beta‐parameter estimate −0.002, CI −0.005 to 0.002 and *P* = 0.375), active CD8 T cells (beta‐parameter estimate −0.003, CI −0.010 to 0.004 and *P* = 0.367), Treg (beta‐parameter estimate −0.007, CI −0.032 to 0.018 and *P* = 0.577), B cells (beta‐parameter estimate 0.0001, CI, −0.001 to 0.001 and *P* = 0.784), NK cells (beta‐parameter estimate 0.0003, CI −0.0004 to 0.001 and *P* = 0.420), ILC1 (beta‐parameter estimate 0.312, CI −1.074 to 1.698 and *P* = 0.649) and ILC3 (beta‐parameter estimate 0.482, CI −0.429 to 1.393 and *P* = 0.289) – were not significantly associated with LCI (Table 2).

### Computational methodologies indicate CRTh2^high^ ILC2 population increased in cord blood from babies with a worse lung function at 6 weeks

To investigate the association between CRTh2^high^ ILC2 in cord blood and lung function parameters (tPTEF/tE% and LCI) in an unbiased way, we employed computational methodologies [t‐distributed stochastic neighbour embedding (tSNE) and PhenoGraph algorithms].

**Figure 3:**
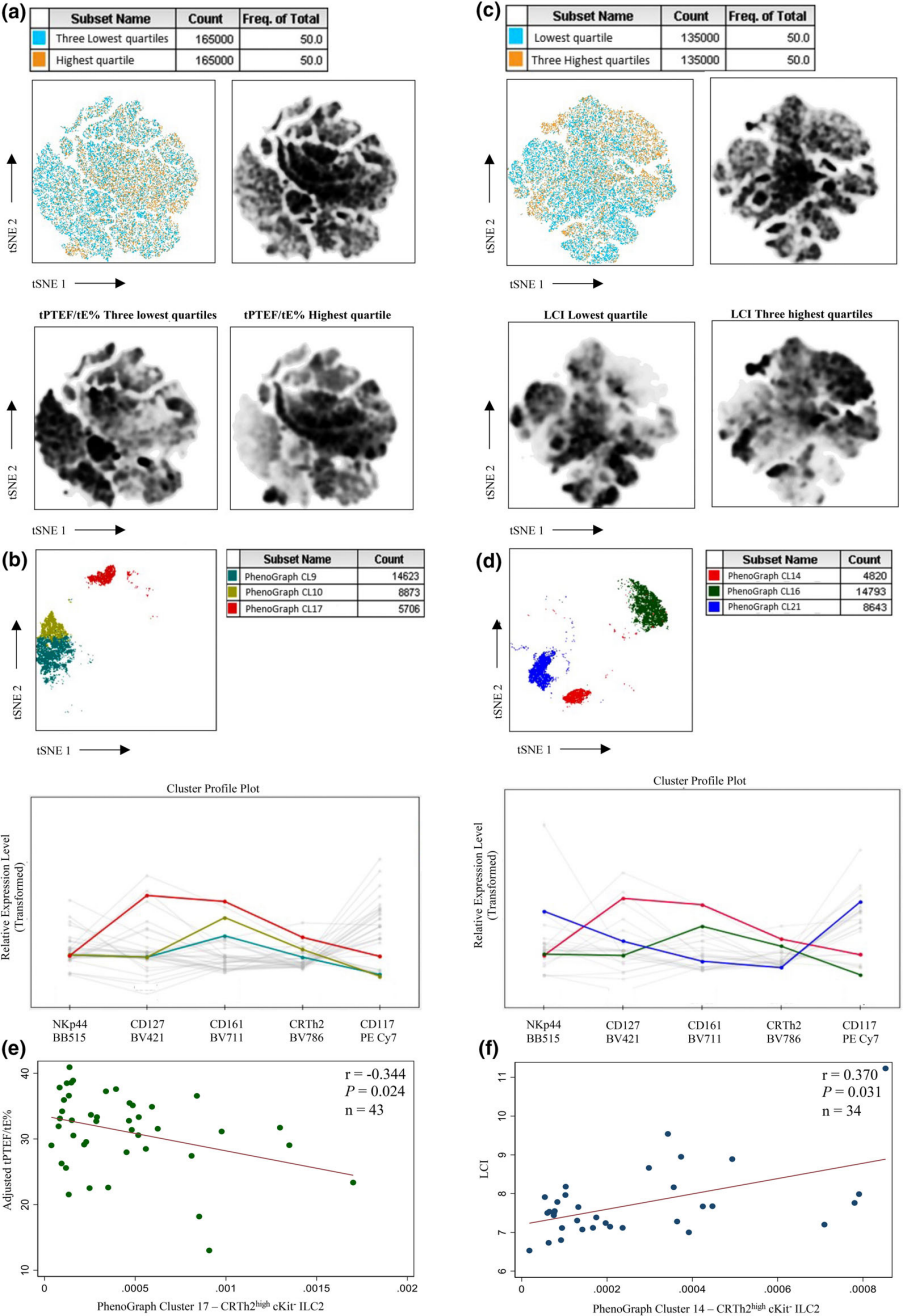
Cord blood ILC2 analysed by computational methods. Visualised tSNE map of cord blood samples, pseudocolour plot and density plots facilitates the visualisation of clusters between the three lowest quartiles of tPTEF/tE% and its highest quartile **(a)** as well as the lowest quartile of LCI and its three highest quartiles **(c)**. Clusters profile plots from the three lowest quartiles of tPTEF/tE% **(b)** and the three highest quartiles of LCI **(d)**. Correlation analysis between adjusted tPTEF/tE% at 6 weeks of age and CRTh2^high^ ILC2 (cluster 17) in the cord blood identified by tSNE and PhenoGraph algorithms **(e)**. Correlation analysis between LCI at 6 weeks of age and ILC2 CRTh2^high^ (cluster 14) in cord blood identified by tSNE and PhenoGraph algorithms **(f)**. Data are expressed as positive cells in 10^3^ of CD45^+^ cells.

Lung function results for both parameters were analysed separately in quartiles with the quartile with the best lung function compared to the three quartiles of lower lung function. We have previously demonstrated that lung function at 6 weeks of age is reduced in babies born to asthmatic mothers.^38^ Thus, in this cohort of infants born to asthmatic mothers, the lung function in the top quartile of infants equates with those above the median in previous normative cohorts.^25^ tSNE plots allowed the visualisation of distinct cluster distribution between lung function groups for tPTEF/tE% (Figure 3a) and LCI (Figure 3c). To explore the hypothesis that cell populations are differentially distributed between the groups defined by lung function quartiles, a PhenoGraph algorithm was applied.^39^ Clusters were individually analysed with three specific cell groups increased among the three lowest quartiles of tPTEF/tE% (Figure 3b). In a separate analysis, three clusters also had cell count increased among the three highest quartiles of LCI (Figure 3d). The relative expression level for each cell surface marker was assessed for all clusters. In the tPTEF/tE% stratified tSNE and PhenoGraph analysis, cluster 17 was commensurate with the typical ILC2 lineage expressing CD127, CD161 and high CRTh2 (Figure 3b and Supplementary figure 1b). Likewise, in the LCI‐stratified tSNE and PhenoGraph analysis, cluster 14 had these same characteristics (Figure 3d and Supplementary figure 1c). With both measures of lung function, these cells were markedly reduced in the quartile with the best lung function.

### Computational methodologies confirm CRTh2^high^ ILC2 as predictors of tPTEF/tE% and LCI in a multivariable and univariable regression

To determine whether CRTh2^high^ ILC2 identified by unbiased computational methodologies present the same characteristics previously observed by manually gated CRTh2^high^ ILC2, linear regression was performed. Clusters 17 and 14 were predictors of tPTEF/tE% and LCI, respectively (Table 3).

**Table 3:** Linear regression analysis used to identify differences between clusters defined by tSNE and PhenoGraph algorithms and its association with tPTEF/tE% and LCI

tPTEF/tE%	Multivariable analysis^a^				LCI	Univariable analysis			
Three lowest quartiles	Coefficient	SE	*P*‐value	95% CI	Three highest quartiles	Coefficient	SE	*P*‐value	95% CI
Cluster 9	0.228	0.187	0.231	−0.152; 0.607	Cluster 14 (CRTh2^high^ ILC2)	1.978	0.584	0.002	0.787; 3.168
Cluster 10	0.612	0.407	0.141	−0.214; 1.438	Cluster 16	0.010	0.012	0.418	−0.015; 0.035
Cluster 17 (CRTh2^high^ ILC2)	−11.237	4.911	0.028	−21.207; −1.266	Cluster 21	−0.015	0.034	0.668	−0.084; 0.054

^a^ Multivariable analysis for each cell type adjusted for male sex, birth order, exclusive breastfeeding until test day, weight gain until test day, and age at test (days).

Correlation analysis using these clusters also matched that of manually gated CRTh2^high^ ILC2 cells and showed an inverse correlation between adjusted tPTEF/tE% at 6 weeks of age and CRTh2^high^ ILC2 (Cluster 17; *r* = −0.344 *P* = 0.024; Figure 3e). There was also a positive correlation between LCI at 6 weeks of age and CRTh2^high^ ILC2 (Cluster 14) identified by tSNE and PhenoGraph algorithms (*r* = 0.370 *P* = 0.031; Figure 3f). These data demonstrate that higher levels of CRTh2^high^ ILC2, which were characterised by two distinct analysis methodologies, were associated with worse lung function.

### CRTh2^high^ ILC2 positively correlates with IL‐5 and IL‐5/IL‐10 ratio

Interleukin‐5 and IL‐10 cytokines were also measured in cord blood plasma, and CRTh2^high^ ILC2 correlated positively with IL‐5 (*r* = 0.281 *P* = 0.008) and the IL‐5/IL‐10 ratio (*r* = 0.251 *P* = 0.019). However, it is conceivable that the correlation between foetal CRTh2^high^ ILC2 numbers and total IL‐5 in cord blood is weakened by maternally derived IL‐5 crossing the placental barrier. Additionally, CRTh2^high^ ILC2 correlated with both eosinophils and other ILC subtypes in cord blood (Table 4).

**Table 4:** Spearman correlation coefficients and *P*‐values for all types of cells

	Eosinophils		Neutrophils		Active T CD4		Active T CD8		Treg cells		B cells		NK cells		ILC1		ILC3	
	*r*	*P*	*r*	*P*	*r*	*P*	*r*	*P*	*r*	*P*	*r*	*P*	*r*	*P*	*r*	*P*	*r*	*P*
Eosinophils																		
Neutrophils	−0.719	< 0.0001																
Active T CD4	0.045	0.675	−0.074	0.487														
Active T CD8	0.070	0.513	−0.011	0.921	0.599	< 0.0001												
Treg cells	−0.018	0.864	−0.011	0.920	0.379	0.0002	0.220	0.036										
B cells	0.098	0.357	−0.220	0.036	0.315	0.002	0.310	0.003	0.132	0.211								
NK cells	−0.214	0.042	0.204	0.052	0.031	0.774	−0.025	0.816	−0.061	0.564	0.132	0.213						
ILC1	0.260	0.013	−0.091	0.390	0.072	0.496	0.179	0.090	0.040	0.706	−0.014	0.893	−0.242	0.021				
ILC3	0.243	0.020	−0.155	0.141	−0.066	0.533	0.080	0.451	−0.178	0.092	0.054	0.613	−0.046	0.664	0.407	< 0.0001		
ILC2	0.222	0.034	−0.090	0.396	−0.124	0.241	0.035	0.743	−0.111	0.296	0.009	0.934	−0.030	0.779	0.447	< 0.0001	0.686	< 0.0001
CRTh2^high^ILC2	0.210	0.045	0.083	0.437	−0.144	0.174	−0.009	0.929	−0.136	0.199	−0.009	0.931	−0.027	0.800	0.388	0.0001	0.570	< 0.0001
CRTh2^low^ILC2	0.138	0.192	−0.001	0.994	−0.115	0.279	0.155	0.142	−0.112	0.290	0.030	0.777	−0.029	0.785	0.484	< 0.0001	0.712	< 0.0001

## Discussion

The immunological relationship between mother and foetus has an important role in the development of allergies and asthma in infants. Children born to mothers with moderate‐to‐severe uncontrolled asthma during pregnancy have a higher risk of developing asthma themselves and have more common lung function abnormalities.^40^, ^41^, ^42^, ^43^ Here, we had the unique opportunity to evaluate the immunity of the newborn prior to disease onset. Cord blood samples from babies born to asthmatic mothers were collected and immunophenotyped, representing the *in utero* immune environment. Further, lung function tests were performed in infants at 6 weeks of life which was analysed in relation to the cord blood cell populations. Interestingly, foetal ILC2 numbers with increased expression of CRTh2 were characterised by two distinct analysis methodologies and were associated with a poorer infant lung function at 6 weeks of age measured by either tPTEF/tE% or LCI.

Previous studies have reported the presence and importance of ILCs in cord blood^44^, ^45^, ^46^; however, cord blood CRTh2^high^ ILC2 populations and their relationship with infant lung function had not yet been investigated. Accumulation and activation of ILC2 are considered a key event for many T2 inflammatory diseases.^47^ A recent study has shown that lung ILC2 accumulation in mice in response to systemic IL‐33 delivery is dependent on the CRTh2.^48^ CRTh2 is one of two functional prostaglandin D2 (PGD_2_) receptors involved in allergic and eosinophilic inflammation in animal models and clinical studies.^49^, ^50^, ^51^ The interaction between PGD_2_ and CRTh2 is implicated in allergic inflammation^52^ and plays a key role in the recruitment of ILC2 to the lung.^48^ The PGD_2_/CRTh2 axis is also involved in eosinophil recruitment and activation and has emerged as a potential pathophysiologic factor for allergy and asthma.^53^, ^54^, ^55^ Here, cord blood CRTh2^high^ ILC2 directly correlated with foetal eosinophil numbers. Together this may indicate a role of foetal ILC2 in promoting eosinophilia.

The balance of T1 and T2 cytokines in pregnancy is thought to be crucial to maternal tolerance of the infant.^56^, ^57^ For this reason, it can be difficult to interpret the skewing towards a T2 immune phenotype and subsequent development of allergic diseases.^58^, ^59^, ^60^, ^61^ However, experimental models have demonstrated a role of maternal IL‐5 in airway function by promoting foetal lung eosinophilia.^13^, ^14^ Interestingly, in the present study we observed associations between cord blood IL‐5, CRTh2^high^ ILC2 numbers and reduced lung function at 6 weeks. Alternatively, higher CRTh2^high^ ILC2 numbers and reduced lung function at 6 weeks may be a surrogate marker for the strong predisposition conferred by maternal asthma to develop asthma and wheeze in childhood. Extensive future experimental and clinical studies are warranted to further dissect the potential relevance of this immune pathway in predicting, and possibly shaping, airway function in early life.

Notwithstanding a lack of mechanistic insights, our findings are of potential clinical significance as asthma in childhood has previously been associated with below‐median tPTEF/tE% as early as the first 3 days of life.^19^ Others have shown lung function in early life tracks throughout life^23^, ^62^, ^63^, ^64^ and can be a risk factor for subsequent development of chronic diseases such as asthma and chronic obstructive pulmonary disease (COPD) and asthma/COPD overlap syndrome.^21^, ^23^, ^65^

A common limitation of studies utilising cord blood is the restricted sample size, and this study is no exception. However, the advantages of being able to access a suitable quantity of blood so early in life have allowed cord blood studies with relatively small sample sizes to make significant contributions to our understanding of early life immune and respiratory development.^66^, ^67^, ^68^, ^69^, ^70^, ^71^ Here, the lowest quartile was used to identify those with lower lung function as previously described.^23^ However, the weakness of this approach is the low lung function group sizes of 8–10 individuals, introducing a sensitivity to selection bias. Notwithstanding the significant investment of time and resources in both the cord blood immunophenotyping and the infant lung function testing, it would be ideal to repeat this finding in a larger cohort to confirm this report. It will also be of interest to determine whether these associations persist to lung function later in life as predicted or whether the associations regress to the mean as the children grow. The inclusion of infants born to asthmatic mothers at high risk to develop impaired lung function in infancy, and wheeze and asthma in later life, may have enabled us to identify associations with smaller case numbers. As there are very few studies to date reporting ILC2 cells in cord blood, it is difficult to appreciate how variable they are across the population but given the magnitude of changes in lung function parameters for every 10^3^ CRTh2^high^ ILC2 of 11 and 1.98 for tPTEF/tE% and LCI, respectively (Table 3), it is highly likely that these levels are physiologically relevant.

In summary, this is the first study to link cord blood CRTh2^high^ ILC2 populations to lower infant lung function. We propose that further mechanistic studies are now required to elucidate the role of foetal ILC2 in shaping the immune response, and how this pathway may be associated with lung function and respiratory outcomes in later life.

## Methods

### Study design and participants

Pregnant asthmatic women, 18 years or older, with asthma diagnosed by a physician and symptoms of asthma or use of asthma therapy (β_2_‐agonist, ICS) in the past 12 months were enrolled in the BLT.^37^ The BLT is a multicentre [Brisbane (QLD), Canberra (ACT), Newcastle (NSW) and Sydney (NSW)] randomised controlled trial of asthma with prospective infant follow‐up. Drug or alcohol dependence, chronic oral corticosteroid use, chronic lung disease other than asthma, concomitant chronic illness were considered as exclusion criteria. Eligible mothers from the BLT cohort at Newcastle who consented to participate in the infant follow‐up had cord blood collected after the baby's birth (*n* = 91) and had lung function performed at approximately 6 weeks of age between March 2017 and November 2019. Sulphur‐hexafluoride MBW (SF6‐MBW) and tidal breathing flow‐volume loop (TBFVL) were performed and LCI (*n* = 34) and tPTEF/tE% (*n* = 43) were assessed, respectively (Figure 1).

### Ethics statement

This research was approved by the Hunter New England Human Research Ethics Committee of the Hunter New England Local Health District (ref no 12/10/17/3.04), and all women provided written informed consent before participation.

### Cord blood collection

Cord blood samples from BLT participants were collected at John Hunter Hospital (New South Wales, Australia) immediately after birth by needle puncture of the umbilical vein after the umbilical cord was detached from the infant. All samples were transferred into EDTA tubes to be processed by a trained staff within 6 h during day and night.

### Infant lung function testing

All children from the BLT cohort whose parents consented to participate in the infant follow‐up were seen at approximately 6 weeks for lung function tests. Lung function was performed on unsedated infants during quiet natural sleep^72^, ^73^ with Exhalyzer D (Eco Medics AG, Durnten, Switzerland). Recordings were based on the operators' experience plus observation of the displayed signals in order to ascertain that (1) the breathing pattern is regular, stable and representative for that infant; (2) there is no trend in instantaneous respiratory frequency (fR); and (3) the signals are technically acceptable (no leaks, artefacts or excessive volume drift). Once the infant has adapted to the mask and is sleeping quietly and breathing regularly, tidal breathing was recorded in epochs of 30 ± 60 s.^26^ All lung function tests on the infants were safe and non‐invasive and performed at the Paediatric Respiratory Laboratory based at John Hunter Children's Hospital (New South Wales, Australia).

### Tidal breathing flow‐volume loop

Tidal breathing flow‐volume loop (TBFVL) was performed, and respiratory rate, tidal volume, minute ventilation and mean tidal inspiratory and expiratory flow were assessed. Tidal breathing recordings started at least 30 s after the initial mask placement. Tests were analysed using WBreath software (v 3.28.0 – Medizintechnik AG, Zurich, Switzerland) and were considered acceptable if more than 30 consecutive breaths (free of sighs, respiratory pauses, irregular volume breaths or air leak) according to international guidelines^26^ and previous studies.^74^, ^75^ The main parameter taken from TBFVL was the time to peak tidal expiratory flow divided by the total time of tidal expiratory flow (tPTEF/tE%). According to the normative data created by the Bern infant lung development cohort, chosen because of similarities in methodology and characteristics of the sample, babies born from non‐asthmatic mothers had the mean tPTEF/tE% of 34.9.^17^, ^76^

### Sulphur‐hexafluoride multiple breath washout

Multiple breath washout testing was performed supine, using an infant mask (size 0, 0/1 and 1; Homedica AG, Huenenberg, Switzerland), according to the ERS/ATS standards of lung function testing of infants,^26^, ^77^ and mask size dead space was corrected during analysis. The flow was measured using an ultrasonic flow meter (Spiroson®; EcoMedics AG, Durnten, Switzerland). A gas mixture containing 4% inert sulphur‐hexafluoride (SF6) gas combined with 21% oxygen with the balance being nitrogen^78^ was the one used following recent recommendation for the measurement of MBW.^28^ The washout period began after a 10‐breath equilibrium period obtained at the end of the tracer gas wash in. The recordings were defined as acceptable for analysis if they occurred during quiet sleep with no apparent volume drift, defined as a change of < 3 mL·s^−1^, no sighs [defined as a marked increase (at least double) in tidal volume with no other artefacts present], within 10 breaths of the wash‐in plateau or 10 breaths after the SF6 concentration has returned to baseline, or 1/40th of the concentration at the start of washout. Tests were analysed using the Wbreath software (v3.28.0. Ndd Medizintechnik, AG, Zurich, Switzerland). Flow and volume were corrected to body temperature, ambient temperature and pressure during data analysis.^75^ From the 34 participants who attended infant lung function appointment at 6 weeks old, four of them had one valid MBW measurement, and 30 of them had two or more valid MBW measurements.

### Flow cytometry staining

Cord blood cells were stained in whole blood, and subsets were predefined based on specific surface markers as follows: eosinophils (CD45^+^, CD193^+^, CD16^−^), neutrophils (CD45^+^, CD193^−^, CD16^+^), B cells (CD14^−^, CD3^−^, CD19^+^), natural killer (NK) cells (CD14^−^, CD3^−^, CD56^+^, CD16^+^), lymphocytes TCD4 cells (CD3^+^, αβT‐cell receptor (TCR)^+^, CD4^+^, CD25^+^, CD127^+^), lymphocytes TCD8 cells (CD3^+^, αβTCR^+^, CD8^+^, CD25^+^, CD127^+^), regulatory T (Treg) cells (CD3^+^, αβTCR^+^, CD4^+^, CD25^+^, CD127^−^), ILC type 1 (ILC1 ‐ CD45^+^, lineage‐negative cells (Lin^−^; CD3, TCR‐αβ, TCR‐γδ, CD19, CD11c, CD94, CD14, CD1a, CD34, CD123, CD303, FcεRIα), CD127^+^, CD161^+^, CD117^−^, CRTh2^−^, NKp44^−^), ILC type 2 (ILC2; CD45^+^, Lin^−^, CD127^+^, CD161^+^, CRTh2^+^, CD117^−^) and ILC type 3 (ILC3; CD45^+^, Lin^−^, CD127^+^, CD161^+^, CRTh2^−^, CD117^+^, NKp44^−/+^; Supplementary table 1). After 30 min of incubation, red blood cells were lysed using BD FACS™ Lysing Solution and washed. Samples were stored at 4°C and acquired on LSRFortessa X‐20 flow cytometer (BD Biosciences, San Diego, CA, USA). For the eosinophil and neutrophil panel, the B‐cell and NK cell panel and the TCD4 and TCD8 panel, a total of 1 000 000 events were acquired and recorded for each subject. The ILC panel had a total of 2 500 000 events recorded for each subject.

### Flow cytometry data analysis

Analyses were done with FlowJo software (v 10.5 ‐ Flow Jo LLC, Ashland, OR, USA) for all cell populations. Results are shown as positive cells in 10^3^ of CD45^+^ cells (for eosinophils, neutrophils and ILCs) and as positive cells in 10^3^ of CD3 (for B cells, NK cells, active CD4 T cells, active CD8 T cells, Treg). To avoid the pitfalls from manually gating, robust computational methods were utilised. This approach was chosen because of the interest in rare subpopulations hard to be certainly identified by standard biaxial gating.

### t‐distributed stochastic neighbour embedding algorithm

The tSNE algorithm, defined as a nonlinear dimensionality reduction approach that embeds the data from high‐dimensional space into a lower‐dimensional map based on similarities^79^ placing similar cells to nearby points, was applied. Prior to tSNE algorithm being applied, populations were gated on forward scatter (FSC) properties from which doublets were excluded based on area versus height parameters of FSC. CD45^+^ and lineage‐negative (CD3, TCR‐αβ, TCR‐γδ, CD19, CD11c, CD94, CD14, CD1a, CD34, CD123, CD303, FcεRIα) cells were previously selected to reduce potential bias in the identification.^80^ The final gate was randomly downsampled and the number of events in each sample normalised to an equal number of cells in each group of interest, allowing an unbiased analysis in the same number of cells. All populations were combined into one .fcs file by concatenating the downsampled populations.

Both lung function parameters assessed were analysed separately and added as additional parameters in the concatenated file making it possible to pull apart individual samples representing different conditions. Lung function results for both parameters were analysed separately in quartiles. The quartile with the best lung function results was compared with the others. For this study, all tSNE analyses were performed on the concatenated sample and the compensated channels that were not used for gating were assessed under tSNE settings: Interaction 1000, Perplexity 30.

### PhenoGraph algorithm

To improve the population analysis, the PhenoGraph algorithm was exploited. PhenoGraph employs a two‐step approach that overcomes some of the limitations of tSNE. It is an unbiased clustering algorithm that automatically detects cell subpopulations.^39^, ^81^ It constructs a *k* nearest‐neighbour graph capturing the phenotypic relatedness of the high‐dimensional data and then applies a graph partition algorithm called Louvain^82^ to dissect the nearest‐neighbour graph into phenotypically coherent subpopulations.^39^ For this purpose, PhenoGraph was also run on the concatenated population using an input of *k* nearest neighbours of 30. Each cluster generated by PhenoGraph was individually investigated. To improve analyses and characterisation of cell populations and to better interpret cluster results in an unbiased way, the ClusterExplorer plugin was used. ClusterExplorer creates summary plots based on the computation method previously used for clustering. In this study, the clustering method was PhenoGraph. Clusters were excluded from further analysis if (1) the absolute cell count was below 4500 cells; (2) clusters were classified as negative for all surface markers; (3) clusters were classified as positive for only one surface marker. The combined PhenoGraph subpopulations identified were then visualised using tSNE^83^ to reduce the dimensional data.^39^ The frequency of cells was assessed using FlowJo software.

### Cytokine determination

Cord blood was added in a LeucoSep® tube with 3 mL of Lymphoprep solution (Lymphoprep™ Fresenius Kabi Norge – Axis‐Shield) to be centrifuged. Plasma was separated and stored at −80°C until further analysis. IL‐5 and IL‐10 were detected simultaneously using cytokine bead array human‐enhanced sensitivity master buffer kit (BD Bioscience), and samples were diluted 1:3. The range of detection was 274–200 000 fg mL^−1^ for each cytokine. Samples were acquired on a FACSCantoII flow cytometer (BD Biosciences) and analysed using FCAP array™ software (v 3.0 – BD Biosciences).

### Statistical analysis

Analyses were performed using Stata IC 16.1 (Stata Corporation, College Station, TX, USA). The Spearman correlation was used to assess relationships between cell types. The influence of confounders was assessed by performing uni‐ and multivariable regression analysis. In multivariable analyses performed to identify variables associated with tPTEF/tE%, the following known confounders were included the following: (1) male sex, (2) birth order, (3) exclusive breastfeeding until test date, (4) weight gain from birth until test date and (5) age at lung function test in days.^38^ In further analyses, tPTEF/tE% was adjusted by these known confounders. To identify variables associated with LCI, a univariable analysis was performed after certifying that LCI was not affected by body size. Pearson analyses were performed to assess correlations with adjusted tPTEF/tE%, and Spearman analyses were performed to assess LCI correlations. For all analyses, statistical significance was considered when *P* < 0.05.

## Conflict of interest

The authors declare no conflict of interest.

## Author contributions

PGG, VEM and JM conducted BLT pregnancy study; PGG, VEM, AMC, PDR and PDS conducted BLT infant follow‐up; JM supervised BLT infant follow‐up; AMC conceived the project and supervised flow cytometry and with JM lung function analyses; PDGB performed and analysed infant lung function; JM, PDR and AMC established lung function protocol; MS, AMC and PMH established flow cytometry panel; WK reviewed statistical analysis; and GMCG and AMC worked on study design. GMCG was involved in data collection, primary author of the manuscript, processed cord blood samples and performed flow cytometry analysis; GMCG and AMC wrote the draft manuscript; and all authors edited the final version of the manuscript.
