# Supplementary material for: Cord blood group 2 innate lymphoid cells are associated with lung function at 6 weeks of age
Source: Clin Transl Immunology. 2021 Jul 21;10(7):e1296. doi: 10.1002/cti2.1296 (PMC8292948; doi:10.1002/cti2.1296)
Supplement: Supplementary file 1 [file CTI2-10-e1296-s001.docx]

**Title: Cord blood group 2 innate lymphoid cells are associated with lung function at six weeks of age**

Gabriela Martins Costa Gomes^1^, Patricia de Gouveia Belinelo^1^, Malcolm R Starkey^1,2,3^, Vanessa E Murphy^1^, Philip M Hansbro^2,4^, Peter D Sly^5^, Paul D Robinson^6^, Wilfried Karmaus^7^, Peter G Gibson^2,8^, Joerg Mattes^1,9^, Adam Collison^1*^

Supporting information

**Supplementary table 1**. Antibodies used for flow cytometry analysis

| CD Antigen | Other Names | Isotope | Clone | Company | Brief Description |
| --- | --- | --- | --- | --- | --- |
| CD1a | HTA1 | PE | HI149 | BD bioscience | Lipid Antigen presentation |
| CD3 | T3 | BV510 | UCHT1 | BD bioscience | TCR co-receptor (Tcell marker) |
| CD3 | T3 | PE-Cy7 | SK7 | BD bioscience | TCR co-receptor (Tcell marker) |
| CD4 | T4 | APC | RPA-T4 | BD bioscience | TCR co-receptor (Tcell marker) |
| CD8 | T8 | APC-H7 | SK1 | BD bioscience | TCR co-receptor (Tcell marker) |
| CD11c | p150 | PE | B-ly6 | BD bioscience | Adhesion |
| CD14 | LPS R | PE | M5E2 | BD bioscience | R for complex of LPS and LBP, innate immune response |
| CD14 | LPS R | PerCP Cy5.5 | M5E2 | BD bioscience | R for complex of LPS and LBP, innate immune response |
| CD16 | FcγRIIIA | BV421 | 3G8 | BD bioscience | Low affinity Fcγ receptor, mediates phagocytosis and ADCC, degranulation |
| CD19 | B4 | PE | HIB19 | BD bioscience | BCR Coreceptor, signalling |
| CD25 | IL-2Rα | PE | M-A251 | BD bioscience | IL-2 receptor α chain |
| CD34 | gp105-120 | PE | 581 | BD bioscience | Adhesion |
| CD45 | LCA | APC | HI30 | BD bioscience | Activation, signalling |
| CD45 | LCA | APC Cy7 | 2D1 | BD bioscience | Activation, signalling |
| CD56 | NCAM1 | PE-Cy7 | B159 | BD bioscience | Glycosylated adhesion protein |
| CD94 | Kp43 | PE | HP-3D9 | BD bioscience | CD94/NKG2A inhibits NK function, CD94/NKG2C activates NK |
| CD117 | cKit | PE-Cy7 | 104D2 | BD bioscience | Signalling, crucial for HSC, gonadal and pigment stem cell growth and development |
| CD123 | IL-3Rα | PE | 9F5 | BD bioscience | IL-3 receptor α chain |
| CD127 | IL-7Rα | BV421 | HIL-7R-M21 | BD bioscience | IL-7 receptor α chain |
| CD161 | NKR-P1A | BV711 | DX12 | BD bioscience | NK cytotoxicity, induces immature thymocytes proliferation |
| CD193 | CCR3 | PE | 5E8 | BD bioscience | Leukocytes chemotaxis, HIV-1 coreceptor |
| CD294 | CRTh2 | BV786 | BM16 | BD bioscience | Regulates immune and inflammatory responses |
| CD303 | BDCA-2 | PE | 201A | Biolegend | Type II transmembrane glycoprotein |
| CD336 | NKp44 | BB515 | p44-8 | BD bioscience | NK activation |
| TCR-αβ | TCR-αβ | PE | T10B9 | BD bioscience | T cell receptor αβ chain |
| TCR-αβ | TCR-αβ | BV510 | IP26 | BD bioscience | T cell receptor αβ chain |
| TCR γδ | TCR γδ | PE | 11F2 | BD bioscience | T cell receptor γδ chain |
| TCR γδ | TCR γδ | FITC | 11F2 | BD bioscience | T cell receptor γδ chain |
| FcεRiα | FcεRIα | PE | AER-37 (CRA-1) | Biolegend | High affinity IgE receptor |

CD Cluster of differentiation, APC Allophycocyanin, APC Cy7/H7 Allophycocyanin cyanine 7, BB brilliant blue, BV brilliant violet, FITC Fluorescein isothiocyanate PE Phycoerythrin, PE Cy7 Phycoerythrin cyanine 7, PerCP Cy5.5 Peridinin-chlorophyll-protein cyanine 5.5. BD Biosciences, San Diego, CA, USA. Biolegend, San Diego, CA, USA

**Supplementary table 2.** Percentage of cells staining positive for CD45 and CD3 in cord blood from babies born to asthmatic mothers for groups which had FACS analysis completed; FACS analysis and acceptable tPTEF/tE% or LCI at six weeks of age

|  | CB Samples – FACS Analysis  n = 91 |  | CB Samples – FACS and tPTEF/tE%  n = 43 |  | CB Samples – FACS and LCI  n = 34 |
| --- | --- | --- | --- | --- | --- |
| % CD45^+^ Cells |  |  |  |  |  |
| Eosinophils | 8.27 |  | 8.28 |  | 8.34 |
| Neutrophils | 68.68 |  | 68.64 |  | 68.82 |
| ILC1 | 0.05 |  | 0.05 |  | 0.04 |
| ILC2 | 0.13 |  | 0.13 |  | 0.14 |
| CRTh2^high^ ILC2 | 0.06 |  | 0.06 |  | 0.06 |
| CRTh2^low^ ILC2 | 0.07 |  | 0.07 |  | 0.07 |
| ILC3 | 0.08 |  | 0.08 |  | 0.08 |
|  |  |  |  |  |  |
| % CD3^+^ Cells |  |  |  |  |  |
| Active T CD4 | 7.93 |  | 7.35 |  | 7.55 |
| Active T CD8 | 2.05 |  | 1.89 |  | 1.97 |
| Treg cells | 5.02 |  | 4.98 |  | 5.03 |
| B cells | 72.06 |  | 72.02 |  | 73.70 |
| NK cells | 51.91 |  | 52.11 |  | 53.08 |

FACS Fluorescence-activated cell sorting, tPTEF/tE Ratio of time to reach peak tidal expiratory flow to total expiratory time, LCI Lung clearance index

**Supplementary figure 1**. **Flow cytometry gating strategy**. Cord blood samples stained and acquired with a LSRFortessa X-20 flow cytometer and analysed using FlowJo software. Analysis based on standard biaxial gating considered CRTh2^high^ ILC2 as CD45^+^, Lin^-^ (CD3, TCR-αβ, TCR-γδ, CD19, CD11c, CD94, CD14, CD1a, CD34, CD123, CD303, FceRIa) CD127^+^, CD161^+^, CRTh2^+^; CRTh2^high/low^ ILC2 **(a)**. According to the gating strategy and ILC2s identified in **(a)** clusters from PhenoGraph algorithm were displayed on a biaxial dot plot, cluster 17 **(b)** and cluster 14 **(c)**. CD45^+^ cells and Lin^-^ cells previously selected.

**
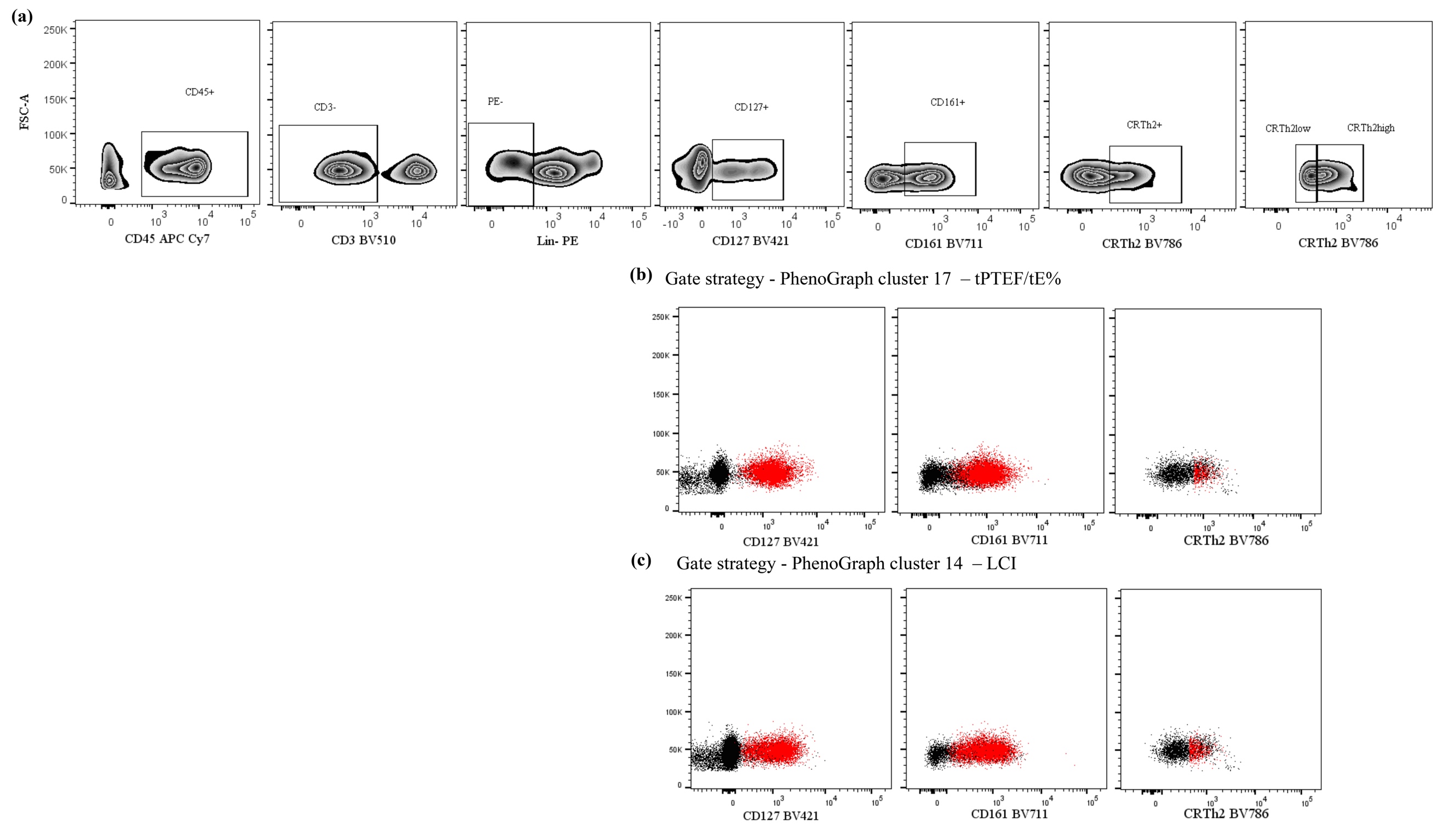
**
